# Iron deficiency anemia, population health and frailty in a modern Portuguese skeletal sample

**DOI:** 10.1371/journal.pone.0213369

**Published:** 2019-03-07

**Authors:** Samantha M. Hens, Kanya Godde, Kristin M. Macak

**Affiliations:** 1 Department of Anthropology, California State University Sacramento, Sacramento, California, United States of America; 2 Department of Sociology and Anthropology, University of La Verne, La Verne, California, United States of America; 3 Department of Sociology and Criminal Justice, Old Dominion University, Norfolk, Virginia, United States of America; University of Alabama, UNITED STATES

## Abstract

**Introduction:**

Portugal underwent significant political, demographic and epidemiological transitions during the 20^th^ century resulting in migration to urban areas with subsequent overcrowding and issues with water sanitation. This study investigates population health during these transitions and interprets results within a framework of recent history and present-day public health information. We investigate skeletal evidence for anemia (cribra orbitalia and porotic hyperostosis) as indicators of stress and frailty–i.e., whether the lesions contribute to susceptibility for disease or increased risk of death.

**Methods:**

The presence and severity of skeletal lesions were compared against known sex and cause of death data to investigate potential heterogeneity in frailty and the relationship between lesions and risk of dying over time. Additionally, we tested for the presence of selective mortality in our data (i.e., whether or not the sample is biased for individuals with higher frailty). Our sample derives from a large, documented, modern Portuguese collection from Lisbon and is the first study of its kind using a documented collection. The collection represents primarily middle-class individuals.

**Results and conclusions:**

Analyses indicated that porotic hyperostosis became more common and severe over time, while cribra orbitalia severity increased over time. Neither process was linked to cause of death. However, there was a significant relationship to sex; males exhibited a higher prevalence and severity of lesions and increased mortality. A Gompertz function showed decreased survivorship in early life but increased survivorship over age 60. Using comorbidities of anemia, we were unable to detect selective mortality–i.e., in our sample, lesions do not represent a sign of poor health or increased frailty and are not significantly linked with a decreased mean age-at-death. However, lesion prevalence and severity do reflect the socioeconomic processes in urban Lisbon during the 1800s and 1900s and the possibility of water-borne parasites as the contributing factor for iron deficiency anemia.

## Introduction

Skeletal samples provide information about the age-at-death, health, and lifestyles of past populations and may be useful in illuminating trends in morbidity and mortality across sociopolitical or socioeconomic transitions throughout history. The 20^th^ century in Portugal was characterized by significant changes to the political-economy resulting in migration into urban centers with subsequent overcrowding and water contamination issues. Thus, an analysis of skeletal indicators of disease (e.g., iron deficiency anemia) coupled with age-at-death data provide an opportunity to investigate whether skeletal lesions are a sign of overall frailty, susceptibility to disease and/or an increased risk of death during this critical period in Portuguese history. However, over twenty-five years ago, the landmark paper, *The Osteological Paradox*: *Problems of Inferring Prehistoric Health from Skeletal Samples* [1], outlined three fundamental problems underlying interpretations of health and disease in past populations. These problems include: (1) demographic nonstationarity, wherein skeletal collections derived from cemetery contexts may reflect populations that were subject to migration or changes in fertility rates; (2) hidden heterogeneity in frailty, wherein individuals exhibit unequal (and ultimately unknown) susceptibility to disease and risk of death due to genetically determined biological differences, differential exposure to disease vectors, or differences in nutritional status owing to behavioral, cultural, or environmental factors; and (3) selective mortality, wherein the skeletal samples represent biased representatives of the living populations from which they are drawn because individuals with the highest frailty at a particular age are more likely to die and enter the skeletal assemblage. While nonstationary populations are a concern for paleodemographers and population-level studies within paleopathology, hidden heterogeneity in risk and selective mortality affect ancient health research more directly [2]. Wood and coworkers [1] argue that researchers must exercise caution when interpreting skeletal lesions and their link to health. Skeletal lesions have traditionally been viewed as a sign of poor health; however, because some skeletal lesions take considerable time to form, they may actually indicate a healthier individual, one who has survived for an extended length of time [3], an idea that is essential to Wood and coworkers’ [1] arguments.

A small number of papers successfully incorporate a research design that directly address these issues [4–12], including selecting an appropriate archaeological context by focusing on simple sites or simple societies, emphasizing subadults as non-survivors, linking frailty and demography, and highlighting lesion formation processes. Appropriate research design should include focusing on severity data over simple prevalence data, identifying diseases that contribute to mortality, and examining disease patterns against age structure [2, 10].

Anemia is a pathological condition characterized by a lack of iron in the blood, usually due to insufficient dietary intake or blood loss due to parasitic infections (e.g., worms). Iron deficiency affects more than 2 billion people worldwide [13]. Iron metabolism is a complex process with a number of contributing factors including the ability of the intestinal cells to adjust to available stores of dietary iron as well as physiological demands. The hormone hepcidin, synthesized in the liver, adjusts iron levels. Hepcidin expression increases with systematic inflammation or infection and tends to be low in girls and young women [13]. Additionally, during pregnancy, infancy, and childhood, the body requires higher levels of iron to support physiological changes. Blood loss due to heavy menses is a well-known cause of iron-deficiency anemia [13]. The prevalence of iron deficiency in menstruating and pregnant women between 1990 and 2010 was approximately 38% [14–15] globally, indicating that women of reproductive age are most at risk of the condition. Furthermore, because iron deficiency has been associated with inflammatory conditions [13], it may predispose an individual to infections and heart failure [16]. Anemic patients show greater than average comorbidities in a modern clinical office [17], with simultaneous presence of anemia with hypertension, hypothyroidism, chronic kidney disease, malignancies, rheumatologic disorders, congestive heart failure, and coronary artery disease.

When iron levels fall below what the body requires, the red marrow is stimulated in an effort to generate a greater number of red blood cells and replenish oxygen levels to tissue [18–22]. This process causes an increase in bone marrow resulting in expansion of the cranial diploë, especially apparent in the cranial vault and the orbital roofs. The compact bone of the outer table eventually resorbs if red blood cell levels are corrected, creating porotic lesions apparent on the ectocranial surface [22].

Skeletally, porotic hyperostosis (PH) manifests as numerous porous lesions in the outer table of the cranial vault, especially the parietal and occipital bones, along with expansion of the marrow cavity (i.e., marrow hypertrophy); while cribra orbitalia (CO) similarly affects the orbital roofs [3, 21]. Modern clinical studies of iron-deficiency anemia [19], epidemiological studies of porotic hyperostosis and cribra orbitalia [23], and radiographic evidence for cranial vault hypertrophy support the link between cranial lesions and anemia [24].

However, some research suggests these cranial lesions have a more complicated etiology beyond simple dietary iron deficiency, to include megaloblastic anemia, environmental insult, parasitism, genetic predisposition, inflammation, and scurvy [18, 21, 22, 25–28]. Parasitic infections are closely linked with sedentism, agriculture, animal domestication, as well as aspects of environment, sanitation, personal hygiene, education, poverty, and economic structures [29]. Emerging research in parasitism is challenging earlier views of the role of iron in health and infection and has implications for the significance of understanding porotic hyperostosis and cribra orbitalia in the balance of diet and disease [3, 30]. However, Oxenham and Cavill [31] and McIlvaine [32] argue that iron-deficiency anemia remains a plausible candidate for lesions attributed to PH and CO and any dismissal of iron deficiency as an underlying cause for these lesions is premature.

Regardless of a unifying catalyst, porotic hyperostosis and cribra orbitalia appear as correlated indicators in the skeletons of individuals who have endured compromised conditions, whether they be tied to nutrition, sanitation, or infectious disease. Thus, linking back to *The Osteological Paradox* [1], these cranial lesions may be a sign of higher frailty and related to an increased risk of death for affected individuals. Bioarchaeologists have documented PH and CO in prehistoric and historic contexts worldwide and commonly use both conditions to assess health and nutritional status on a population scale. Studies cover an array of causal mechanisms including dietary-based deficiencies [27, 33–36], weaning [37], parasitic causes [27, 28], and pathogen load [21], across an array of geographic regions (e.g., Australia and the Pacific Islands [38], Greece [39], China [36] and Mexico [40]). However, no PH or CO studies have directly addressed the issues of hidden heterogeneity or selective mortality and no archaeological skeletal samples had documentation on the individuals comprising the collection. Additionally, insight into modern public health issues may be gained from the examination of skeletal lesions in documented historic populations, which may lead to a better understanding of health trends across historic and modern contexts.

This study utilizes a research design intended to maximize understanding of the potential relationship between PH and CO lesion incidence and severity with sex, known cause of death, and over time (as measured by year of birth and year of death). Using known comorbidities to anemia, we also test for the presence of selective mortality in our sample by examining whether the presence of lesions (i.e., the assumption that more lesions indicates increased frailty) contributed to the risk of death when compared to individuals without lesions. Using a large, documented cemetery of modern Portuguese comprising both subadult and adult individuals, an age-specific survivorship function is applied to find corresponding parameters in subpopulations that inform the impact of selective mortality and individual heterogeneity in the risk of death and disease. Then, we can evaluate whether (1) there are more lesions or more severe lesions in certain subgroups that might be traditionally expected to show higher levels of anemia and subsequently higher frailty (e.g., adult females), (2) lesions are linked to known cause of death, and (3) year of birth or year of death affects lesion prevalence. The use of a documented collection provides a level of detail unobtainable from an archaeological collection. Finally, we interpret our lesion data within the demographic, socioeconomic, and public health context of the time represented by the sample and to documented anemia prevalence in living adult Portuguese [41].

### Background of the sample and associated portuguese history

This study used the Luís Lopes Collection of identified modern Portuguese skeletons curated at the National Museum of Natural History and Science in Lisbon, Portugal (formally known as the Boçage Museum). The collection comes from three cemeteries in Lisbon (Alto de São João, Benfica, and Prazeres) dating to the 19^th^ and 20^th^ centuries. Documentation for interred individuals includes sex, age-at-death, birth and death dates, occupation, and cause of death.

Tradition predicates the exhumation of individuals from temporary graves after five years or complete skeletonization to allow for reuse of gravesites. If family fail to claim the remains or to pay the fee for *ossorio* storage, remains are destroyed via incinerator and included in a communal grave. Beginning in 1981, the National Museum stepped in to collect and curate unclaimed remains and individuals for whom fees were not paid [42]. All available remains were acquired until 1991, which resulted in a collection composed of predominantly adult remains as many subadults were directly interred in communal graves [43]. Cardoso [43] reports more than half of the infants and children were interred in the communal grave at Alto de São João from at least 1875 [44] and possibly into the 20^th^ century, particularly those from a lower socioeconomic status (SES).

Most of the individuals represented are of middle SES from the city of Lisbon, a conclusion supported by occupation data and the method of acquisition of the remains [42]. However, many individuals may have been born outside Lisbon and immigrated to the city where they eventually passed [45]. The middle class is predominantly represented as the cost of temporary interment is prohibitive for the lowest SES and the wealthy are usually able to pay for permanent interments [43]. While the collection provides a cross-section of middle SES individuals, some higher SES individuals are in the collection, as evidenced by their occupation and interment in the cemetery associated with wealthier residents (Prazeres) [43]. Moreover, in the collection, earlier dates of death are associated with a higher SES [43].

Common male occupations include service and sales workers, skilled workers, craftsmen, and similar jobs. Female occupations are most frequently recorded as housewife, maid, teacher, or student. The socioeconomic context at the time of burial was dominated by agriculture until 1900, when industrial growth emerged in Lisbon. The expansion the domestic market occurred slowly, with low productivity in all branches of textiles, metalworking, and food production. As the main port city, Lisbon increased more rapidly in industrial production [42, 46].

The first half of the 20^th^ century experienced accelerated urban growth due to large migrations of rural farmers into cities in search of work, resulting in overcrowding and poor living conditions for the working class and highly impoverished [45]. For the most underprivileged members of society, such as the sick, poor, widows, and orphans, the family was still the basis for support and survival. As a result of urbanization, migration increased the disease load of city inhabitants wherein overcrowding coincided with water sanitation issues. Stratification of classes punctuated the period, whereby the middle class was subsumed into the elite and the working class morphed into a peasantry. Nutritional disparities are evident between port cities and inland towns and the negative effects of urbanization have been documented in various Portuguese skeletal collections from this time period, including increased mortality rates and decreased stature [42].

## Materials and methods

### Data collection

This study examined the skeletal remains of 540 individuals from the Luis Lopez Collection of identified, modern Portuguese individuals curated at the National Museum of Natural History and Science in Lisbon, Portugal. Remains are available for study to qualified individuals with permission. Specimen catalog numbers, all demographic information and lesions scores for individuals used in this research are listed in the S1 Appendix. In our sample, year of birth ranges from 1806 to 1950, and year of death ranges from 1880 to 1970. Sexes were approximately equal in representation and include 281 females (52% of total population) and 259 males (48% of total population). The demographic profile by age and sex is presented in Table 1.

**Table 1:** Age and sex distribution for individuals in the study. Adult age groups align with the EMPIRE study of the living residents of Portugal [41, 47].

Age Group	# Males	# Females
0–10	10	7
11–17	8	7
18–24	13	16
25–34	21	14
35–44	19	11
45–54	58	29
55–64	35	37
65–79	63	101
80+	32	59
Total	259	281

The presence and severity of porotic hyperostosis and cribra orbitalia were visually assessed by the third author (KMM). All skulls showing preservation of the relevant regions (i.e., cranial vault and superior eye orbits) were scored for lesions. Presence/absence and severity of expression were recorded on a scale of 0–3 according to the procedures for data collection outlined by the codebook for the Global History of Health Project, modified from the Western Hemisphere Project [48]. Use of the codebook provides uniformity in scoring lesions and standardization in method to support replicability of results, objective identification of lesions, and is the foundation for making an evidence-based diagnosis of lesions. Lesions need only be present in one orbit or one portion of the cranial vault in order to generate a cumulative porosity score for the individual [48]. If only one orbit or one parietal exhibited lesions and the other did not, the highest score assigned was used for this study. These cases represent a small percent of the total sample and are retained in the study. A small number of individuals had only one side of the cranium present for observation; the present side was scored and included in the analysis. There were 59 separate causes of death listed in the formal death registration records. These records are reliable and have a great degree of accuracy, as they were generated from death certificates [42]. Cause of death (COD) categories contained as few as 1 individual (asthma), to a maximum of 82 individuals (tuberculosis). These were grouped into four categories representing degenerative, infectious, neoplastic, and other conditions following other published studies examining population health [49, 50] (Table 2). Some of these may have no association with anemia, while others may be comorbidities (discussed below). The “other” category was designed to capture causes of death that were not degenerative, infectious, or neoplastic [50], and is not expected to be related to frailty or anemia; but is included for comparison. These four COD categories were used for subsequent analyses comparing lesion presence and severity to COD in order to more broadly evaluate the relationship of anemia to COD.

**Table 2:** Groupings for individual cause of death categories (following [49, 50]).

Group	Causes of Death
**Degenerative**	Vascular lesions, heart disease, other heart and circulatory diseases, arteriosclerosis, nephritis, uremia, renal sclerosis, cirrhosis of liver, gastrointestinal ulcers, diabetes
**Infectious**	Tuberculosis (all), syphilis, typhoid, leprosy, meningitis, influenza, pneumonia, bronchitis, septicemia, scarlet fever, plague, polio, smallpox, malaria, diarrhea, colitis, diphtheria, dysentery, meningococcal meningitis
**Neoplastic**	Cancers: gastrointestinal, uterine, lung, brain, prostate, renal, colo-rectal, oral, breast
**Other**	Old age, senility, sudden death, ruptured uterus, gunshot, carbon monoxide poisoning, suicide, anemias, appendicitis, hernia, intestinal obstruction, congenital

In a direct test of the issue of selective mortality, comorbidities with anemia were identified from the medical literature [17, 51] and cross-referenced with documented cause of death in the Portuguese sample. Thus, we test if the comorbidity mortality was selective for individuals with anemia, i.e., whether individuals with lesions showed higher frailty and were more likely to die from comorbid conditions than those without lesions. Comorbidities of anemia [17, 51] identified in this sample include: cancer, cirrhosis of the liver, congenital heart failure, hypertension, chronic kidney disease, diabetes mellitus, chronic obstructive pulmonary disease, and coronary artery disease.

### Statistical analyses

Statistical analyses investigated the interaction between lesion presence and severity against sex and COD in relation to mortality. The time frame represented by this sample spans important epidemiological and demographic shifts in Portugal’s history (see Discussion). Therefore, statistical analyses were conducted to model for changes over time by each parameter (e.g., sex, COD). Both year of death and year of birth were available in the collection’s documentation and so they were both used as variables to represent change over time in the sample. Binomial and ordinal (the latter using a proportional odds ratio) logistic regressions were invoked where CO and PH presence were dependent variables for the binomial logit and CO and PH severity for the ordinal logit. Sex and COD were main independent variables that were tested separately against the dependent variables. Year of death and year of birth were included individually and their effects were held constant as they are confounders in relation to the main independent variables of interest. To summarize, the statistical models were built with one dependent variable (CO or PH presence or severity) and two independent variables (sex or COD as the main independent variable of interest and year of death or year of birth as a confounding variable for time). *P*-values are provided (from applying a z test to the t values in ordinal regression), which reflect associated confidence intervals, and allows for easier comparisons to other studies who use *p*-values generated from statistical tests.

A Gompertz function plotted with a Kaplan-Meier survivorship described the survivorship in the sample. R scripts were modified by the second author (KG) from coding written by Dr. Lyle Konigsberg available on his website (http://faculty.las.illinois.edu/lylek/) to conduct the survivorship analysis. A Gompertz model distribution is typical when demographic sample age cohorts are differentially represented in the sample, such as the Luís Lopes Collection.

Selective mortality and frailty were tested by comparing the frail and non-frail samples to one another, separated by presence of comorbidity. Our selective mortality protocol incorporated a binomial logistic regression in order to test whether the presence of lesions in the comorbidity sample led to frailty, while also controlling for age-at-death and time. The sample type (comorbidity present or not) was the dependent variable and the independent variables were lesion presence (main independent variable), age-at-death (confounder for age-related changes), and year of death or year of birth (confounder for time). Age-at-death, year of death, and year of birth were treated as continuous variables. All statistical analyses were performed using Microsoft Excel (2008) and R Statistical Computing software, version 3.0.2. [52]. Statistical significance was judged using the Bonferroni corrected *p*-value cutoff of 0.025 (calculated as 0.05 divided by the number of statistical tests per hypothesis, in this case two), which mostly eliminates the impact of multiplicity of *p*-values from running multiple tests to explore the same hypothesis (Type I error).

## Results

The mean age-at-death differed between males (53.980 years) and females (61.953 years). A one-way ANOVA showed a statistically significant difference in age-at-death for both sexes (F-value = 8.9512, *p*-value = 0.0029), indicating differences in survivorship in favor of female longevity by approximately eight years.

**Fig 1:**
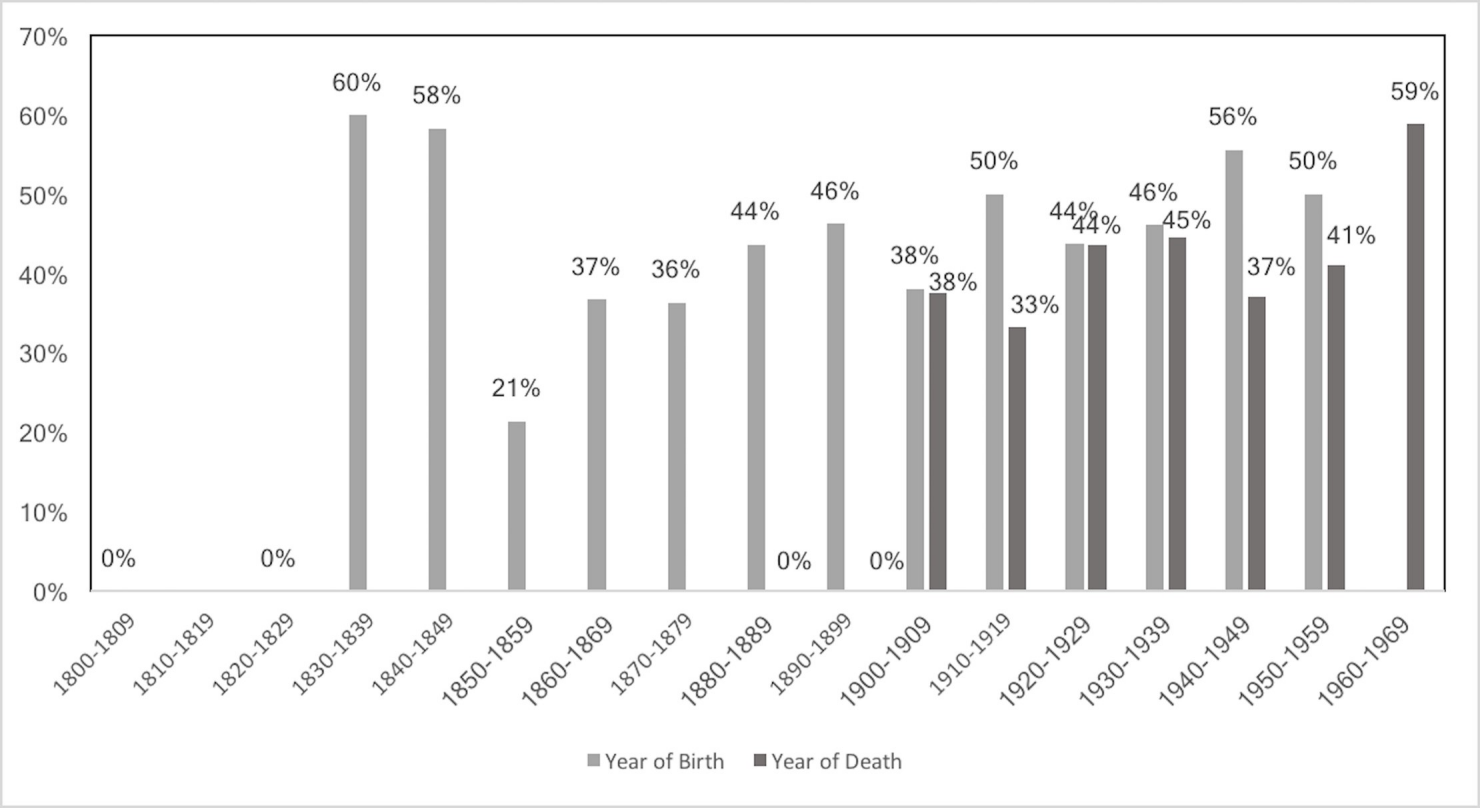
Prevalence of CO by year of birth and year of death.

**Fig 2:**
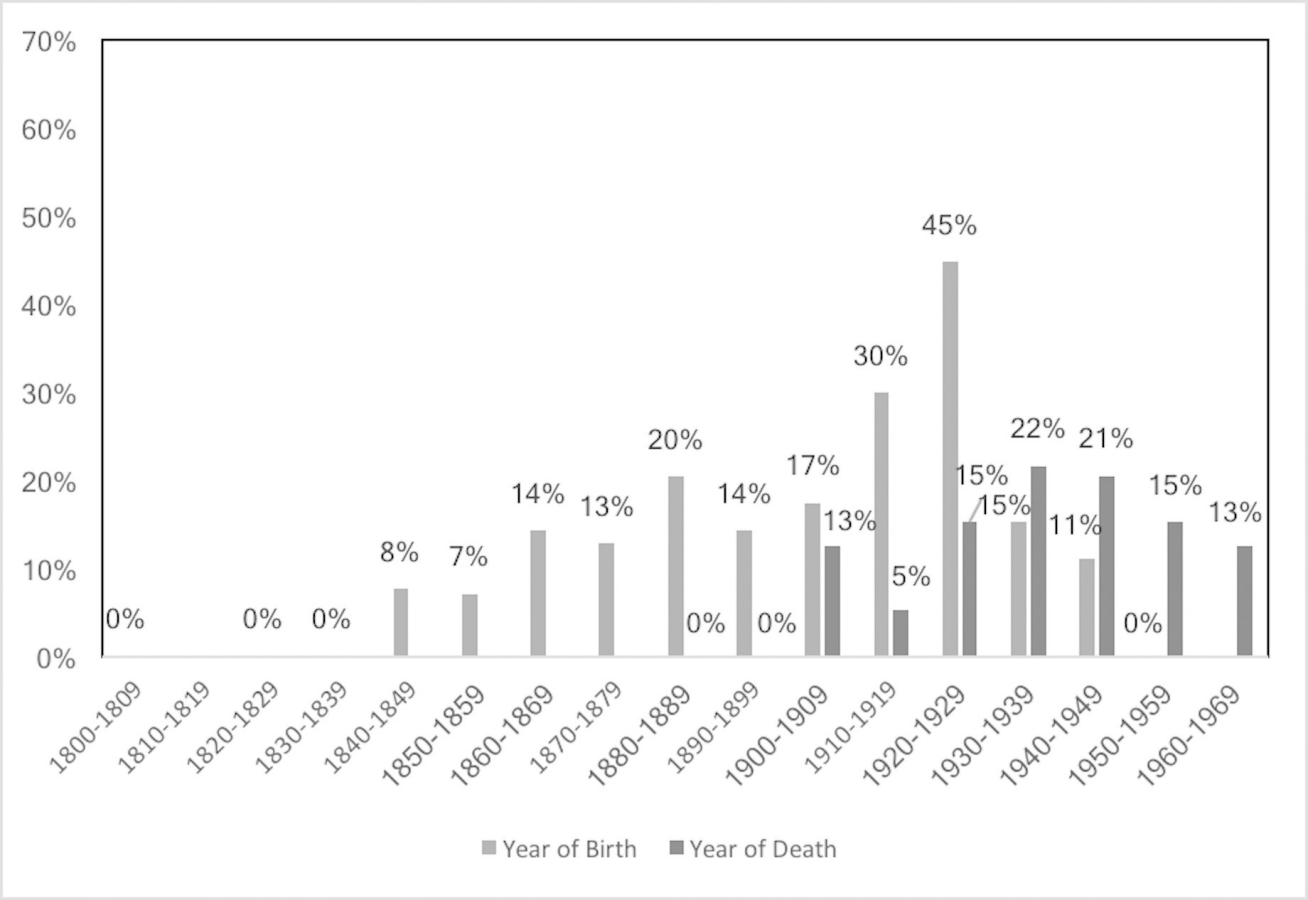
Prevalence of PH by year of birth and year of death.

**Fig 3:**
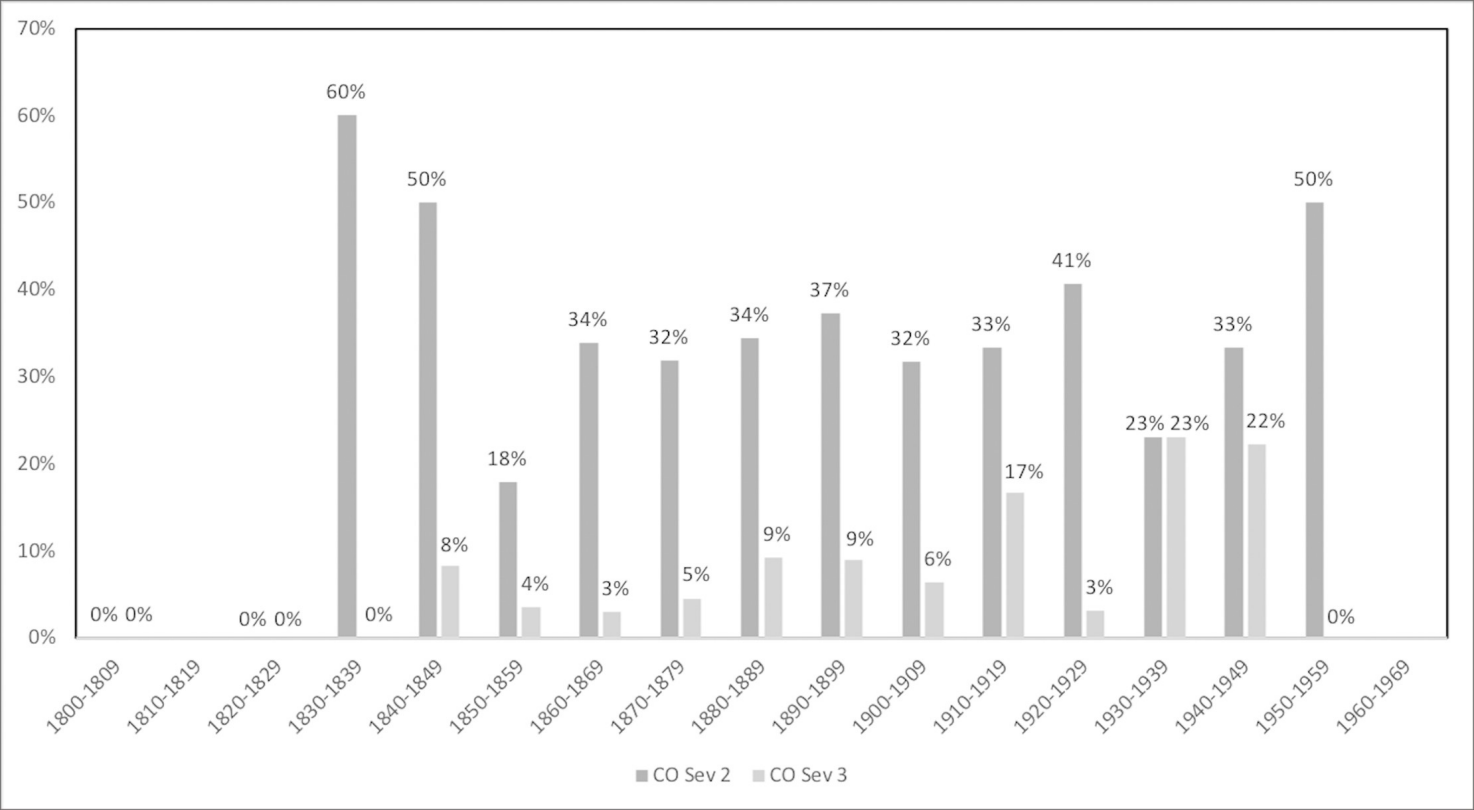
Severity of CO by year of birth. A score of 2 indicates a cluster of fine foramina covering a small area, while a score of 3 indicates substantial areas covered by small or large foramina [48].

**Fig 4:**
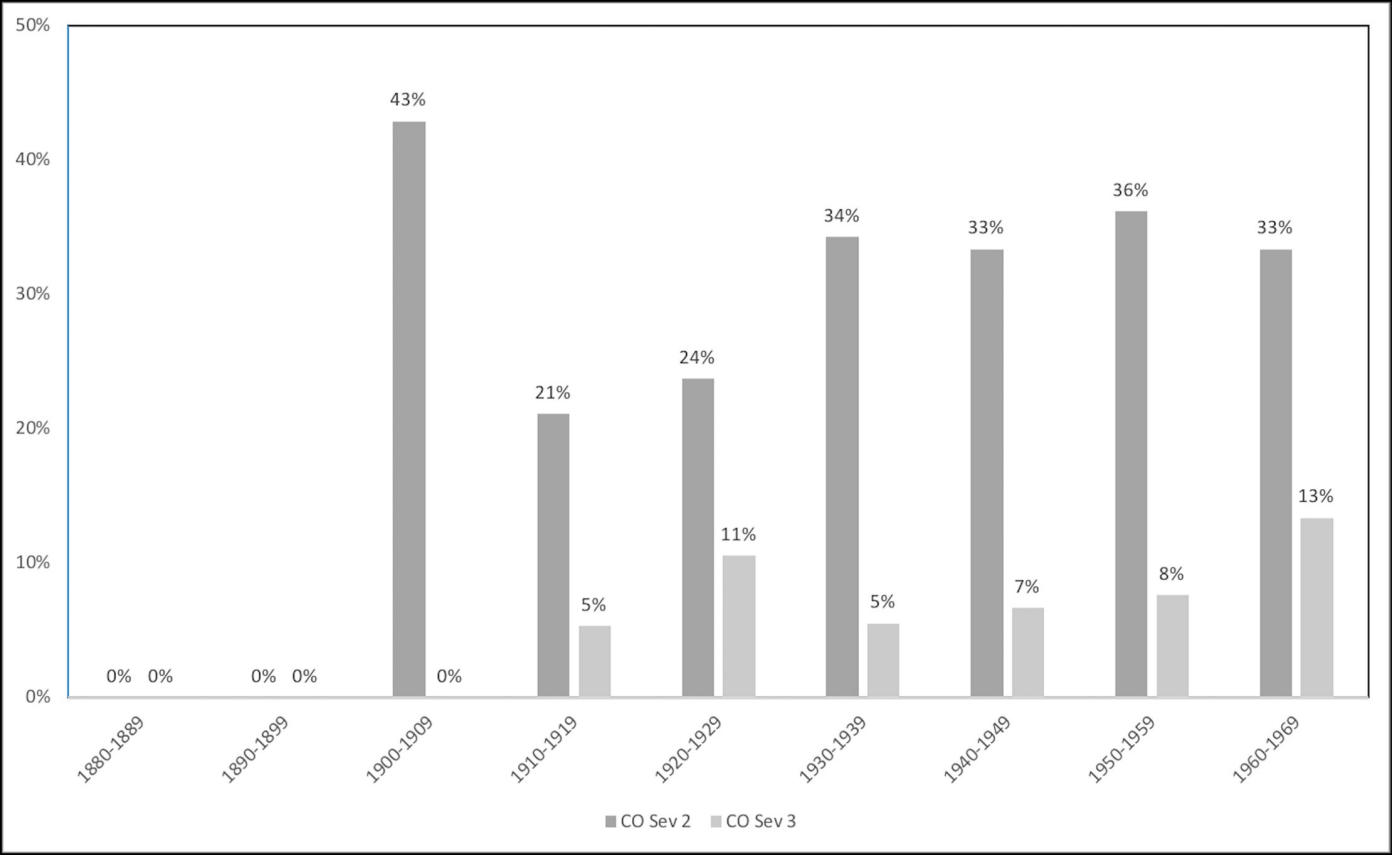
Severity of CO by year of death. A score of 2 indicates a cluster of fine foramina covering a small area, while a score of 3 indicates substantial areas covered by small or large foramina [48].

**Fig 5:**
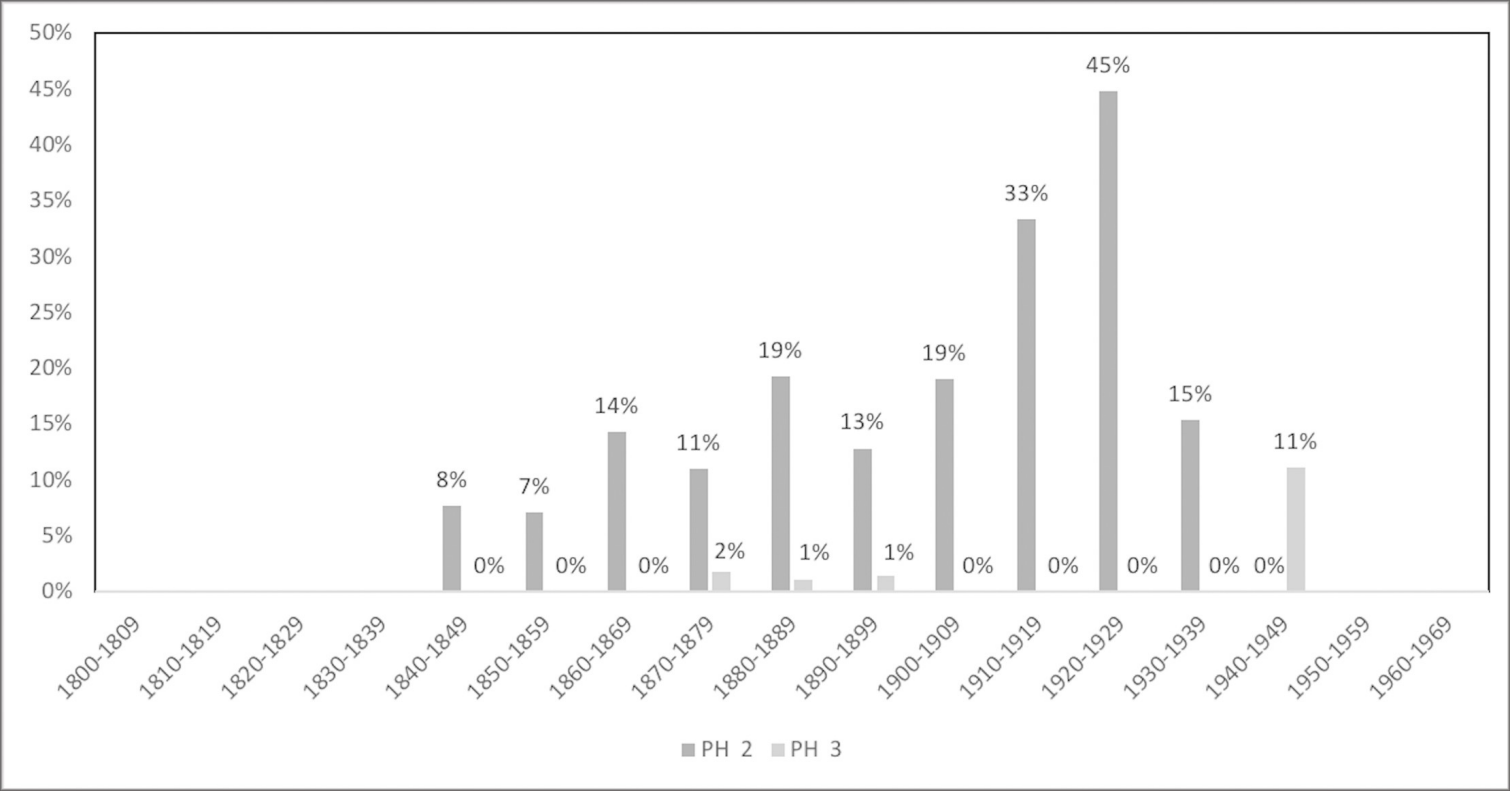
Severity of PH by year of birth. A score of 2 indicates slight pitting or severe porosity, while a score of 3 indicates gross parietal lesion with excessive enlargement of bone [48].

**Fig 6:**
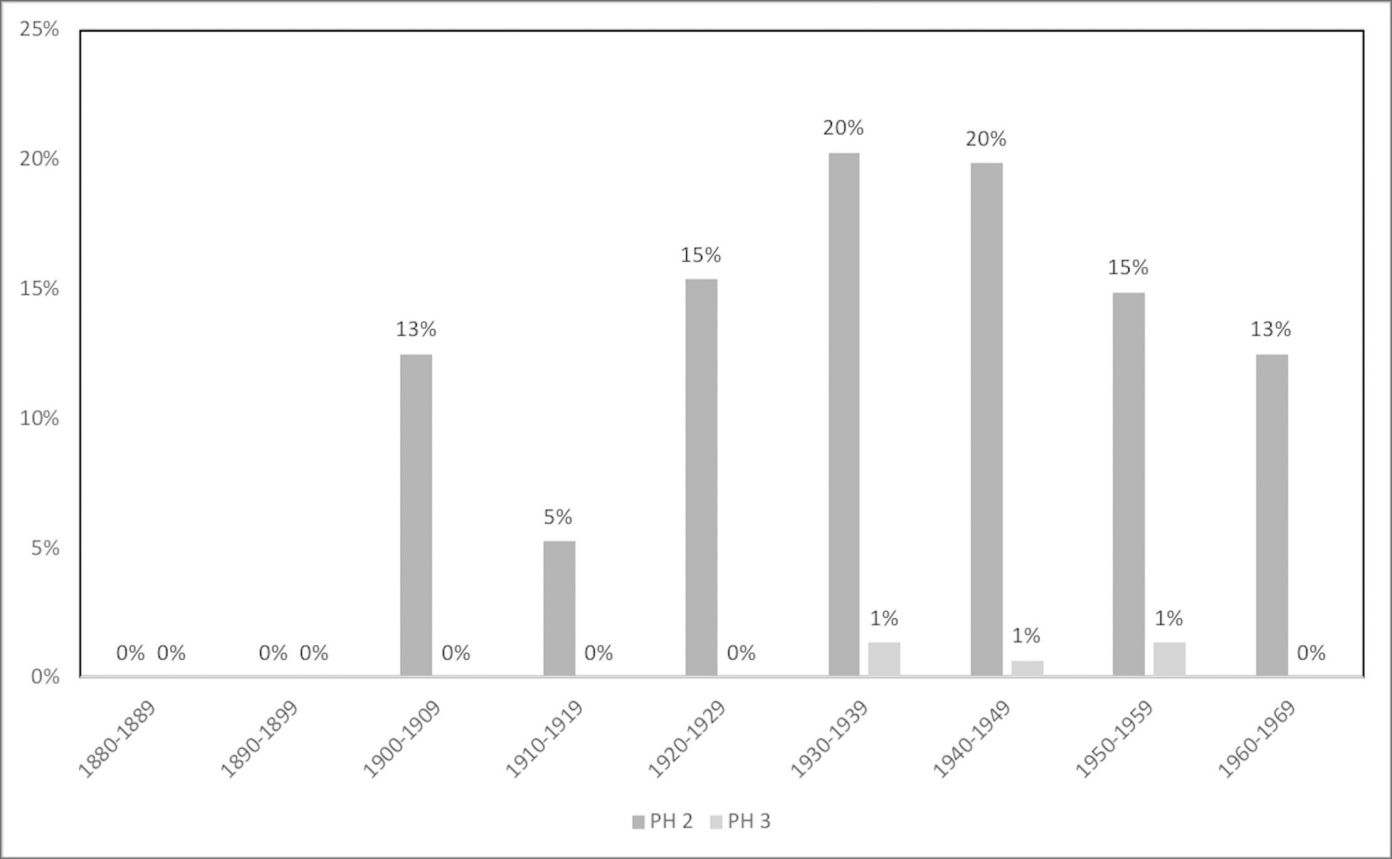
Severity of PH by year of death. A score of 2 indicates slight pitting or severe porosity, while a score of 3 indicates gross parietal lesion with excessive enlargement of bone [48].

The prevalence of PH and CO by sex and age group is shown in Table 3. Additionally, Figs 1–6 depict the prevalence and severity of PH and CO by decade. CO was documented with greater frequency than PH. Some form of lesion was present in approximately half of the sample. Of the 540 individuals scored, a total of 289 individuals displayed no lesions, 160 individuals displayed only CO, and 35 individuals displayed only PH. The highest rates of CO were found in young males, ages 11–24, while young adult and middle-aged adult males exhibited the highest prevalence of PH at ages 18–24 and 35–44.

**Table 3:** Prevalence of PH and CO by sex and age.

Age Group	Male % CO	Female % CO	Male % PH	Female % PH
0–10	30	71	0	0
11–17	75	57	38	14
18–24	77	25	53	31
25–34	48	36	19	29
35–44	26	27	47	18
45–54	45	34	17	17
55–64	37	32	20	3
65–79	31	43	17	15
80+	39	42	13	5

The positive coefficients for time (year of death or birth) were significant in all analyses of sex and COD differences except CO presence, indicating PH presence and severity and CO severity increased over time in our sample (Table 4). Lesion presence/severity varied across sex, when year of death was held constant, in only one test: PH presence, whose coefficient (0.6206) shows PH prevalence in males increased over time as compared to female prevalence (Table 4). Lesion presence/severity did not differ among COD, when time was held constant (Table 5). For selective mortality, the presence of lesions was not increased in either the sample with comorbidities or without (Table 6). Age-at-death and time were both significant in the selective mortality models and the positive coefficients revealed that age-at-death increased in the comorbidity sample, as did year of death/birth. The older ages-at-death were likely more prevalent in the comorbidity sample as it excluded accidental deaths not related to health-induced causes. In sum then, selective mortality was not present in these results, demonstrating lesions did not contribute to frailty for individuals dying of anemia comorbidities.

**Table 4:** Summary statistics from all comparisons between CO and PH to sex, while accounting for time, and using binomial (z values) and ordinal (t values) logistic regression. Values outside of parentheses are when year of death (DY) was held constant and those in parentheses are when year of birth (BY) was held constant.

Model	Independent Variables	Coefficient	z or t value	*p*-value
**CO presence**				
	Sex M	0.0623 (0.0023)	0.3510 (0.0130)	0.7260 (0.9894)
	DY or BY	0.0077 (0.0071)	1.1130 (1.8640)	0.2660 (0.0623)
**CO severity**				
	Sex F	-0.0699 (0.0006)	-0.3999 (0.0032)	0.6893 (0.9974)
	DY or BY	0.0077 (0.0084)	110.3200 (0.0012)	<0.0001* (<0.0001)*
**PH presence**				
	Sex M	0.6206 (0.5297)	2.6400 (2.2230)	0.0082* (0.0262)
	DY or BY	0.0009 (0.0144)	0.1010 (2.8890)	0.9193 (0.0039)*
**PH severity**				
	Sex F	-0.5693 (-0.4734)	-2.3902 (-1.9769)	0.0168 (0.0480)
	DY or BY	0.0020 (0.0153)	28.2728 (197.7400)	<0.0001* (<0.0001)*

* Significant value (*p*<0.025)

**Table 5:** Summary statistics from all comparisons between CO and PH to COD categories, while accounting for time, and using binomial (z values) and ordinal (t values) logistic regression. Values outside of parentheses are when year of death (DY) was held constant and those in parentheses are when year of birth (BY) was held constant.

Indicator	Type of COD	Coefficient	z or t value	*p*-value
**CO presence**				
	Infectious	-0.0349 (-0.2112)	-0.1480 (-0.875)	0.8830 (0.3817)
	Neoplastic	0.2292 (0.2295)	0.7810 (0.7810)	0.4350 (0.4350)
	Other	-0.0899 (0.0877)	-0.3910 (-0.381)	0.6960 (0.7034)
	DY or BY	0.0071 (0.0083)	0.9920 (2.0860)	0.3210 (0.0370)
**CO severity**				
	Degenerative	0.0881 (0.0943)	0.3893 (0.4157)	0.6969 (0.6775)
	Infectious	0.0406 (-0.1481)	0.1731 (-0.6261)	0.8625 (0.5312)
	Neoplastic	0.4392 (0.4282)	1.4909 (1.4507)	0.1359 (0.1468)
	DY or BY	0.0067 (0.0097)	77.8793 (109.4384)	<0.0001* (<0.0001)*
**PH presence**				
	Infectious	0.0781 (-0.1968)	0.2650 (-0.6430)	0.7910 (0.5200)
	Neoplastic	0.0942 (0.0709)	0.2550 (0.1910)	0.7990 (0.8486)
	Other	-0.4856 (-0.5206)	-1.5380 (-1.6340)	0.1240 (0.1023)
	DY or BY	0.0021 (0.0164)	0.2330 (3.1370)	0.8160 (0.0017)*
**PH severity**				
	Degenerative	0.3672 (0.3966)	1.2335 (1.3122)	0.2173 (0.1894)
	Infectious	0.4443 (0.1739)	1.4649 (0.5597)	0.1430 (0.5756)
	Neoplastic	0.4435 (0.4495)	1.1717 (1.1720)	0.2413 (0.2411)
	DY or BY	0.0030 (0.0173)	23.1072 (128.7690)	<0.0001* (<0.0001)*

* Significant value (*p*<0.025)

**Table 6:** Summary statistics from all comparisons between CO and PH to COD categories, while accounting for time, and using binomial (z values) logistic regression. Values outside of parentheses are when year of death (DY) was held constant and those in parentheses are when year of birth (BY) was held constant.

Model	Confounders	Coefficient	z value	*p*-value
**CO presence**		0.1569 (0.1448)	0.7610 (0.7010)	0.4467 (0.4832)
	Age-at-death	0.0239 (0.0595)	4.4700 (5.9730)	<0.0001* (<0.0001)*
	DY or BY	0.0360 (0.0355)	3.7540 (3.8200)	<0.0002* (<0.0001)*
**CO severity**		0.0013 (0.0094)	0.0050 (0.0330)	0.9960 (0.9730)
	Age-at-death	0.0232 (0.0614)	4.2430 (6.0490)	<0.0001* (<0.0001)*
	DY or BY	0.0386 (0.0381)	3.9690 (4.0400)	<0.0001* (<0.0001)*

* Significant value (*p*<0.025)

**Fig 7:**
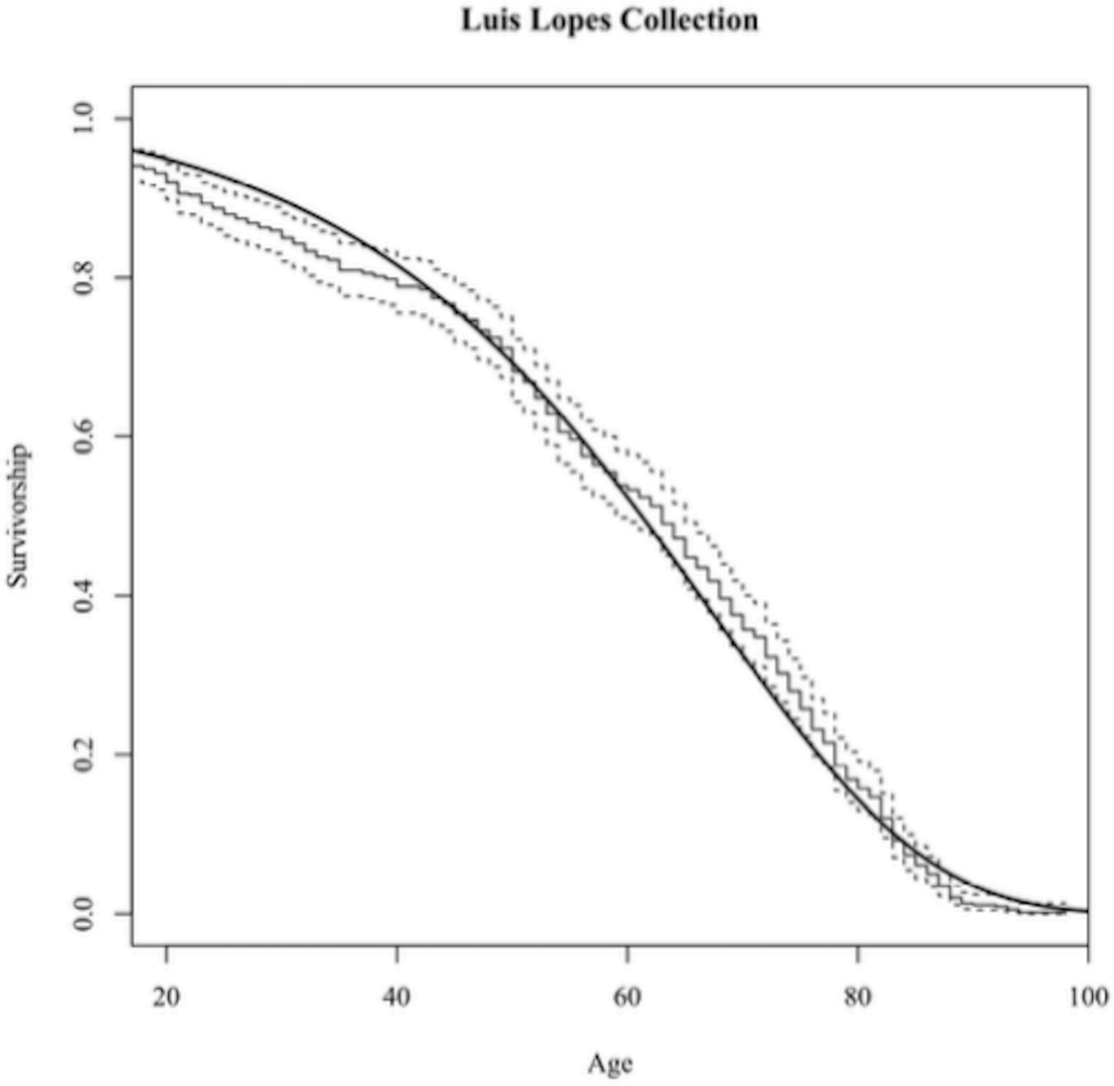
**A Gompertz function (solid line) fit to a Kaplan-Meier survivorship (stepped lines) curve in historic Portuguese**.

A Gompertz hazard model produced parameters of α = 0.001449602 and β = 0.053574250 and appeared to have a good fit to the Kaplan-Meier survivorship (see Fig 7). The survivorship curve produced for the Portuguese collection by the Gompertz function indicates moderate population decline from birth to early adulthood. Survivorship declines steadily from birth to middle age. After age 80, the hazard of dying increases exponentially as survivorship declines.

## Discussion

We explored the relationship between lesions attributed to anemia and sex, cause of death, frailty, and mortality in a modern, documented Portuguese skeletal sample. Because the skeletal sample represents people living in a time of significant socioeconomic change in Portugal, we examined our results across time. Additionally, we formally addressed the issue of selective mortality by testing the differences in mortality patterns between frail and non-frail individuals. Our research design linked frailty to demography, emphasized lesion severity and prevalence, and considered diseases that may contribute to mortality (i.e., comorbidities), while utilizing age-structured data. Numerous authors [2, 10, 53–55], state that this rigor is required to appropriately address the issues raised by Wood and coworkers [1] in *The Osteological Paradox*.

At the outset of this analysis, we expected that females would show more lesions and/or more severe lesions when compared to males. The blood loss due to menstruation, along with pregnancy, places females at a higher risk of anemia worldwide [13–15, 41, 47] and has been documented archaeologically [28, 56]. However, while lesions were common in our sample (>50%), the highest rates of both PH and CO were found in males; but only the presence of PH in males significantly increased over time. Klein [57] discusses the effects of hormones on sex differences in infection and finds that males are more susceptible than females to infections from parasites, fungi, bacteria, and viruses–an issue that may affect parasite-derived iron deficiency. If the anemia in our sample was parasite-based rather than dietary-based, it may explain the higher prevalence of anemic lesions in the males. This male bias is upheld by Fischer’s [58] study of infectious pathogens and Bernin and Lotter’s [59] study of the effects of steroid hormones on infectious disease. However, Klein and coworkers [60] report females at higher risk of morbidity and mortality in influenza outbreaks, especially women of reproductive age. The NIH recommends including sex as a criterion in studies to better understand sex-related differences in infection and disease [61]. While young adult males are particularly affected by lesions in this sample, this may be an artifact of younger females with lesions already being removed from the population at younger ages (see Table 1).

Our second expectation was that if anemic lesions were a sign of poor health and increased frailty, then they may show a relationship to COD. After grouping the myriad causes of death in our sample into four broad categories representing infection, degenerative conditions, cancers, and miscellaneous, we were unable to detect a significant link between lesions and COD groups. Results indicated no relationship between lesion presence or severity and COD in our sample.

Finally, we expected that if selective mortality was present in our sample, individuals with the highest frailty at any given age would be more likely to die compared to their non-frail counterparts (i.e., selective mortality would be present) akin to that reported in other studies [8, 9]. After linking CO and PH with comorbidities identified in the medical literature, we grouped our sample into frail and non-frail subsets, based on the presence of lesions and associated comorbidity. This hypothesis was not supported by our analysis, indicating that selective mortality is not present in our sample; the presence of anemia in individuals whose COD was a comorbidity did not contribute to frailty in our sample. This result may be due to the source of the comorbidities being cause of death; the length of time the comorbidity affected the individual while they also had anemia is unknown. Thus, tests for the presence of selective mortality indicated that the presence of lesions was irrelevant; i.e., lesions did not contribute to frailty for individuals with associated comorbidities nor with people dying from other causes of death. However, lesions did increase with older ages-at-death and became more common over time.

Starting at the beginning of the 20^th^ century and continuing to mid-century, there was significant migration into the city resulting in overcrowding and decreased living conditions in urban environments [45]. This overcrowding led to water sanitation issues [45], and water-borne parasites are a probable cause of iron deficiency anemia–explaining the rise in lesions over time in our sample and exposing the sexes equally. However, as mentioned above, males are at a higher risk of parasitic infection [58, 59] and not surprisingly show higher prevalence of anemic lesions in our sample. Many individuals in our sample likely migrated into urban Lisbon at some point in their life, exposing them to overcrowding and poor water quality as they aged–and lesions did increase with older age-at-death in our sample. Additionally, while our sample is comprised of primarily middle-class individuals, many of the higher-class individuals (i.e., Prazares cemetery) were born to the earliest time periods in our collection when anemia prevalence was lowest. It is entirely possible that the increase in prevalence of anemia might also be an artifact of SES as later births represented primarily middle-class individuals.

A more nuanced discussion of the broader sociopolitical and economic changes occurring across Portugal may be warranted to place our results in historical context. During the time period represented by the Luís Lopes Collection, the country was transitioning from a monarchy to a Republic, followed by a dictatorship, which would have contributed to compromised economic viability of working-class individuals, compromised nutrition, and limited health care options. According to Moreira and Filipa [62], Portugal began epidemiological and demographic shifts starting 1900–1920, with a change point in the 1940s, and persisting until around the 1970s [62]. This shift is evidenced in the overall pattern of disease prevalence in the collection [43] and in the decrease in infectious and parasitic diseases as a COD, among others. The prevalence of infectious and parasitic disease decreased over the period from 1930–1970 (1930 = 21.1%, 1940 = 20.9%, 1950 = 13.5%, 1960 = 1.9%, 1970 = 1%) [62]. Mortality was higher in Portugal (21%) in 1900 compared to Western Europe (17%). The life expectancy for women in Portugal in 1900 was 35.2 years (men = 33.3 years) [63], 60.7 years in 1950 (men = 55.6 years) [63], and just under 83.6 years (men = 77.3 years) in 2012 [62].

The recent decrease in mortality in Portugal has been attributed to sanitation, nutrition, and healthcare changes in the 1960s and 1970s [64]. At this time the number of homes with tap water, showers and/or bathtubs, indoor toilets, electricity, and sewage systems began to increase [65], leading to an increase in sanitation and better hygiene. Nutrition also changed with the incorporation of more milk, meat, fats, sugar, eggs, and alcohol, which led to an increase in caloric consumption [65]. An increase in the number of doctors also began at this time [65]. Heights increased greatly after these changes were implemented [65–67], which reflects better health of the Portuguese. Stature is often used as a suitable proxy for growth, as it is highly dependent on appropriate nutrition and lack of disease [68–71]. However, our sample predates these modern improvements and therefore would be expected to show higher prevalence of disease.

Recent research from the EMPIRE study [41, 47] in public health on nearly 8,000 living residents of Portugal found the overall prevalence of anemia was 19.9%, and that anemia was more common in women (20.8%) and especially pregnant women (54.2%). Additionally, the authors report anemia was more frequent in young adult (18–34) and much older adults (80+) and that there were marked regional asymmetries with anemia more prevalent in Lisbon (39%). Our results were not consistent with the higher rates of anemia in women and older adults reported in the EMPIRE studies; but did find higher rates of anemic lesions in young adults and was consistent with higher prevalence for an urban environment. We found much higher levels of anemic lesions overall in our sample than reported for modern Portuguese. In the EMPIRE study [41] biomarker and survey data from residents were randomly selected so that each inhabitant would have an equal probability of being sampled, regardless of adult age, gender, ethnicity, SES, or nutrition. The Luís Lopes Collection represents a cross-section of middle-class individuals in early historic urban Lisbon where diet and environment-driven stressors were expected to have affected these families.

Before the shift in the 1960s and 1970s, children exhibited stunting due to the living conditions at the time [43, 46, 72] and a lack of antibiotics [72]. The high levels of cribra orbitalia and the increasing prevalence of anemic lesions prior to 1970 coincides with this decreased growth potential and may be linked to the lack of available antibiotics to counter water-borne parasites.

### Conclusion

The Luís Lopes Collection yields complex data that provide a glimpse into past health in Portugal. Selective mortality was not detected in our sample, so lesions did not contribute to frailty for individuals dying from comorbidities. Indeed, individuals with lesions died at older ages than those without lesions, suggesting lesions are a sign of survivorship. There were significant differences in lesion severity and incidence over time and between males and females. Males in the Luís Lopes Collection showed a higher incidence of porotic hyperostosis, while also exhibiting a significantly younger mean age-at-death when compared to females.

The mortality pattern has important implications for our understanding of the living population that produced the study sample. For this study, demographic records denote place of birth for individuals in this sample, which indicate regional movement occurred in the lifetime of this population. City of birth spans the country of Portugal, but all individuals died in Lisbon, which characterizes a population that was neither insular nor stationary. Regional movement of a population over a lifetime impacts both the distribution of pathology in a skeletal assemblage and heterogeneity of risk. Extra-regional migration into Portugal was low at this time and demographic and genetic indicators do not suggest appreciable differences among the regions in Portugal and the population is relatively homogeneous [73]. Therefore, this is not likely a bias reflected in the results here.

If lesions signified poor health and increased frailty, then lesion expression would reflect an elevated mortality hazard. However, if lesions represent survivorship, then lesions may be a sign of lower frailty relative to people without lesions and would not show an elevated mortality hazard. Our results do not support the traditional model for disease–i.e., in our sample, lesions do not represent a sign of poor health or increased frailty and are not significantly linked with a decreased mean age-at-death. But lesion prevalence and severity do reflect the rich history of urban Lisbon during the 1800s and 1900s and the possibility of water-borne parasites as the contributing factor for iron deficiency anemia. We concur with other researchers who stress the importance of sex- and age-structured data as a critical tool for evaluating lesion and mortality data [2, 53, 54]. This study was the first to use a documented skeletal collection to address the issues of hidden heterogeneity in frailty and selective mortality as well as providing important contextual information on local and regional history in our analysis of morbidity and mortality.

## Supporting information

S1 Appendix Catalog numbers for all specimens used in this study from the Luis Lopez collection at the National Museum of Natural History, Lisbon, Portugal (n = 540). (DOCX)
