# Correction: Iron deficiency anemia, population health and frailty in a modern Portuguese skeletal sample

**DOI:** 10.1371/journal.pone.0215235

**Published:** 2019-04-04

**Authors:** Samantha M. Hens, Kanya Godde, Kristin M. Macak

## Notice of Republication

An incorrect version of S1 Appendix was published in error. This article was republished on March 29, 2019 to correct for this error. The article’s Data Availability statement has been updated to reflect this change. Please download this article again to view the correct version.
