# Supplementary material for: Iron deficiency anemia, population health and frailty in a modern Portuguese skeletal sample
Source: PLoS One. 2019 Mar 7;14(3):e0213369. doi: 10.1371/journal.pone.0213369 (PMC6405098; doi:10.1371/journal.pone.0213369)
Supplement: S1 Appendix — (DOCX) [file pone.0213369.s001.docx]

S1 Appendix. Catalog numbers for all specimens used in this study from the Luis Lopez collection at the National Museum of Natural History, Lisbon, Portugal (n=540).

| Cat. # | Cat. # | Cat. # | Cat. # | Cat. # | Cat. # | Cat. # | Cat. # | Cat. # | Cat. # | Cat. # | Cat. # |
| --- | --- | --- | --- | --- | --- | --- | --- | --- | --- | --- | --- |
| 2 | 112 | 236 | 308 | 353 | 414 | 466 | 517 | 665 | 796 | 1052 | 1147 |
| 4 | 115 | 237 | 309 | 355 | 415 | 467 | 518 | 666 | 809 | 1053 | 1149 |
| 6 | 116 | 238 | 310 | 356 | 416 | 468 | 520 | 672 | 820 | 1057 | 1150 |
| 8 | 119 | 239 | 311 | 358 | 418 | 469 | 521 | 673 | 833 | 1058 | 1151 |
| 9 | 127 | 240 | 312 | 360 | 419 | 470 | 522 | 678 | 842 | 1060 | 1155 |
| 10 | 131 | 241 | 313 | 361 | 422 | 471 | 523 | 679 | 847 | 1061 | 1156 |
| 18 | 135 | 242 | 314 | 367 | 423 | 472 | 543 | 681 | 855 | 1064 | 1157 |
| 23 | 136 | 243 | 315 | 368 | 424 | 474 | 545 | 682 | 862 | 1065 | 1158 |
| 30 | 138 | 244 | 316 | 369 | 426 | 475 | 547 | 683 | 893 | 1070 | 1168 |
| 31 | 150 | 245 | 317 | 370 | 427 | 476 | 559 | 684 | 911 | 1071 | 1169 |
| 33 | 152 | 247 | 318 | 371 | 428 | 477 | 564 | 686 | 923 | 1073 | 1172 |
| 34 | 153 | 251 | 319 | 372 | 429 | 479 | 572 | 692 | 944 | 1074 | 1174 |
| 35 | 154 | 253 | 320 | 373 | 430 | 480 | 577 | 703 | 951 | 1078 | 1176 |
| 37 | 156 | 257 | 321 | 374 | 431 | 481 | 578 | 705 | 965 | 1080 | 1177 |
| 39 | 157 | 258 | 322 | 377 | 433 | 482 | 581 | 706 | 966 | 1081 | 1178 |
| 42 | 158 | 261 | 323 | 378 | 435 | 484 | 582 | 708 | 969 | 1083 | 1180 |
| 44 | 163 | 263 | 324 | 380 | 436 | 485 | 583 | 717 | 974 | 1089 | 1181 |
| 48 | 166 | 264 | 325 | 381 | 437 | 486 | 585 | 719 | 977 | 1090 | 1182 |
| 53 | 167 | 265 | 326 | 382 | 438 | 488 | 586 | 725 | 978 | 1091 | 1183 |
| 54 | 176 | 267 | 327 | 383 | 439 | 490 | 587 | 729 | 981 | 1092 | 1185 |
| 56 | 177 | 270 | 328 | 384 | 440 | 491 | 588 | 740 | 984 | 1093 | 1186 |
| 60 | 178 | 271 | 329 | 385 | 442 | 492 | 589 | 747 | 985 | 1095 | 1187 |
| 61 | 181 | 272 | 330 | 386 | 443 | 493 | 590 | 748 | 986 | 1096 | 1189 |
| 62 | 183 | 273 | 331 | 387 | 444 | 494 | 591 | 751 | 988 | 1097 | 1191 |
| 63 | 189 | 274 | 332 | 388 | 445 | 495 | 596 | 753 | 993 | 1098 | 1192 |
| 65 | 190 | 275 | 333 | 389 | 446 | 496 | 597 | 755 | 1003 | 1100 | 1195 |
| 69 | 191 | 276 | 334 | 390 | 447 | 497 | 598 | 759 | 1016 | 1101 | 1196 |
| 70 | 196 | 280 | 335 | 391 | 448 | 498 | 600 | 760 | 1018 | 1104 | 1198 |
| 72 | 198 | 285 | 336 | 392 | 449 | 499 | 602 | 761 | 1022 | 1105 | 1199 |
| 73 | 201 | 287 | 337 | 393 | 450 | 500 | 603 | 765 | 1024 | 1106 | 1200 |
| 74 | 202 | 291 | 338 | 394 | 451 | 501 | 604 | 768 | 1025 | 1109 | 1220 |
| 75 | 203 | 292 | 339 | 396 | 452 | 503 | 605 | 769 | 1029 | 1116 | 1222 |
| 77 | 204 | 293 | 340 | 397 | 453 | 504 | 608 | 770 | 1031 | 1119 | 1226 |
| 80 | 210 | 294 | 341 | 399 | 454 | 505 | 610 | 772 | 1032 | 1120 | 1227 |
| 81 | 212 | 295 | 342 | 400 | 455 | 506 | 611 | 774 | 1033 | 1123 | 1231 |
| 82 | 215 | 296 | 343 | 401 | 456 | 507 | 614 | 775 | 1037 | 1126 | 1233 |
| 83 | 220 | 297 | 344 | 402 | 457 | 508 | 615 | 777 | 1038 | 1127 | 1235 |
| 89 | 221 | 299 | 345 | 404 | 458 | 509 | 619 | 778 | 1039 | 1128 | 1237 |
| 91 | 222 | 300 | 346 | 405 | 459 | 510 | 621 | 780 | 1040 | 1131 | 1238 |
| 92 | 223 | 301 | 347 | 406 | 460 | 511 | 630 | 781 | 1042 | 1133 | 1239 |
| 97 | 224 | 302 | 348 | 407 | 461 | 512 | 632 | 783 | 1043 | 1138 | 1241 |
| 102 | 226 | 303 | 349 | 408 | 462 | 513 | 642 | 786 | 1044 | 1139 | 1242 |
| 104 | 230 | 304 | 350 | 410 | 463 | 514 | 651 | 791 | 1046 | 1141 | 1244 |
| 105 | 232 | 305 | 351 | 411 | 464 | 515 | 654 | 793 | 1048 | 1143 | 1245 |
| 109 | 233 | 307 | 352 | 412 | 465 | 516 | 655 | 795 | 1049 | 1145 | 1246 |
